# The effects of sleep deprivation on the processing of emotional facial expressions in young adults with and without ADHD

**DOI:** 10.1038/s41598-021-93641-7

**Published:** 2021-07-09

**Authors:** Ami Cohen, Kfir Asraf, Ivgeny Saveliev, Orrie Dan, Iris Haimov

**Affiliations:** Psychology Department, Center for Psychobiological Research, Emek Yezreel, Max Stern Yezreel Valley College, Afula, Israel

**Keywords:** Psychology, Diseases, Neurological disorders

## Abstract

The ability to recognize emotions from facial expressions is essential to the development of complex social cognition behaviors, and impairments in this ability are associated with poor social competence. This study aimed to examine the effects of sleep deprivation on the processing of emotional facial expressions and nonfacial stimuli in young adults with and without attention-deficit/hyperactivity disorder (ADHD). Thirty-five men (mean age 25.4) with (*n* = 19) and without (*n* = 16) ADHD participated in the study. During the five days preceding the experimental session, the participants were required to sleep at least seven hours per night (23:00/24:00–7:00/9:00) and their sleep was monitored via actigraphy. On the morning of the experimental session, the participants completed a 4-stimulus visual oddball task combining facial and nonfacial stimuli, and repeated it after 25 h of sustained wakefulness. At baseline, both study groups had poorer performance in response to facial rather than non-facial target stimuli on all indices of the oddball task, with no differences between the groups. Following sleep deprivation, rates of omission errors, commission errors and reaction time variability increased significantly in the ADHD group but not in the control group. Time and target type (face/non-face) did not have an interactive effect on any indices of the oddball task. Young adults with ADHD are more sensitive to the negative effects of sleep deprivation on attentional processes, including those related to the processing of emotional facial expressions. As poor sleep and excessive daytime sleepiness are common in individuals with ADHD, it is feasible that poor sleep quality and quantity play an important role in cognitive functioning deficits, including the processing of emotional facial expressions that are associated with ADHD.

## Introduction

Attention-deficit/hyperactivity disorder (ADHD) is a common neuropsychiatric disorder that includes symptoms of impulsivity, hyperactivity, and inattention. In addition to the well-documented cognitive impairments of ADHD, such as difficulties with attention, reward response, and executive functioning, children and adults with ADHD demonstrate social, interpersonal, and emotional difficulties^1^. In particular, ADHD is associated with deficits in appraisal of others’ emotional state, including difficulties in recognition and labeling of facial expressions of emotion^2–7^.

The ability to recognize emotions from facial expressions is essential to the development of complex social cognition behaviors^8,9^, and impairments in this ability are associated with poor social competence^1^. Individuals with ADHD appear to have particular difficulty in recognizing negative emotions such as fear and anger^2,6,10,11^, and disrupted response inhibition toward facial anger cues^12^.

Processes related to major aspects of attention and cognitive functioning can be measured using the visual oddball paradigm^13,14^, according to which subjects have to maintain vigilance and respond to an infrequent deviant stimulus (i.e., "target") among a series of standard stimuli (i.e., "non-target"). Failures to respond to the target ("omission errors"), slow reaction time to the target (RT), and high variability of reaction time to the target (RT standard deviation [RTSD]) reflect failures in vigilance (sustained attention), while responses to the non-target (commission errors) reflect failure in response inhibition^15^. The oddball paradigm is also used to investigate the special role of emotional content in perception and attention^16^, by employing, for example, neutral facial expressions as the standard stimuli (i.e., "target") and emotional facial expressions as the deviant stimuli (i.e., "non-target"). Using such a paradigm, Raz and Dan^17^ demonstrated that participants with ADHD generally perform worse than controls, as reflected in higher rates of omission errors and commission errors, slower RTs, and higher RTSD. However, higher rates of omissions and slower RTs in the ADHD participants compared with controls were only found in response to face targets but not in response to geometric shape targets, suggesting a specific ADHD-related deficit in the processing of emotional facial expressions.

ADHD is also associated with a greater risk of sleep difficulties. Specifically, 25–50% of children and adults with ADHD experience problems initiating and maintaining sleep^18,19^, which may contribute to their difficulties in vigilance, response inhibition, and other aspects of attention and in their ability to process emotional facial expressions. Lending support to this possibility, sleep deprivation tends to have a negative effect on sustained attention^20–22^ and on the ability to identify emotional expressions^23–25^. Moreover, studies have demonstrated that sleep difficulties aggravate impulsivity and inattentiveness in children and adults with ADHD^26,27^. In controlled studies, sleep restriction or deprivation has been shown to negatively affect attentional capabilities of children^20^ and adults with and without ADHD^22^. However, it is currently unknown whether sleep deprivation affects the attentional process involved in the detection of, and response to, emotional facial expressions in general, and among adults with ADHD in particular.

Thus, the current study aimed to compare the effects of sleep deprivation on the processing of facial expressions and nonfacial stimuli (geometric shapes) between young adults with and without ADHD using the visual oddball paradigm. The oddball task required the participants to face two frequent standard stimuli (a neutral face or geometric shape), and two deviant “target” stimuli (an angry face or geometric shape with a cross in it) that had to be detected as quickly as possible. Based on the previous findings, we hypothesized that performance in the oddball task would deteriorate following sleep deprivation, as reflected in higher omission and commission error rates, slower RTs, and higher RTSD compared to baseline. We expected that this deterioration would be more pronounced in the ADHD group and in response to facial targets. Our second hypothesis was that, prior to sleep deprivation, participants with ADHD would perform worse than controls on the oddball task in general and in response to face targets in particular.

## Methods

### Participants

Nineteen male adults with ADHD (mean age 24.7 years; standard deviation [SD] 5.3 years) and 16 male adults without ADHD (mean age 26.3 years; SD 3.0 years) took part in the study. Seven participants from each study group were college students, recruited via ads placed on campus. The rest of the participants were recruited from the local community using the snowball sampling technique. Only men were recruited to the study in order to reduce variability, given that women's menstrual cycle may influence sleep, alertness, and, thus, cognitive functions related to these factors^28^.

Eligibility criteria for inclusion to the ADHD study group were: (1) six or more symptoms on both the hyperactivity-impulsivity and inattention scales on the ADHD assessment questionnaire (see below)^29^; (2) a prior diagnosis of adult ADHD by a psychiatrist or by a neurologist; and (3) meeting ADHD criteria on the modified adult version of the ADHD module in the Diagnostic Interview Schedule for Children (DISC)^30^.

Eligibility criteria for inclusion to the control study group were: (1) at most three symptoms on each of the hyperactivity-impulsivity and inattention scales on the ADHD Rating Scale-IV; (2) no previous ADHD diagnosis and (3) failure to meet ADHD criteria on the modified adult version of the DISC ADHD module.

Exclusion criteria for both study groups were: (1) use of centrally active medications (except ADHD medications); (2) diagnosis of periodic limb movements in sleep, obstructive sleep apnea, or restless legs syndrome, screened for by using the Mini Sleep Questionnaire^31^ and by interviews; (3) night shift employment; and (4) psychopathology as diagnosed using the Symptom Checklist-90. The reason for the last exclusion criterion was twofold: first, an attempt to isolate the effects of ADHD on the studied measures, and second, an awareness that a 25-h sleep deprivation is a stressful situation which may have a significant negative impact on individuals with serious psychiatric disorders.

The vision of all participants was normal or corrected-to-normal. Among the participants diagnosed with ADHD, the mean age of diagnosis was 16.5 (SD = 7.94), with 64.29% diagnosed prior to the age of 18. Four of the ADHD participants were regular users of stimulant ADHD medications and the remaining participants of this group reported the sporadic use of such medications. All ADHD participants agreed not to use these medications from 24 h before the start of the experiment until its end (use of a washout period for such medications was previously reported; e.g.^32^). Participants who smoked (N = 9) were mostly light to moderate smokers, smoking 10 cigarettes or less per day on average.

The study conformed to the principles outlined in the Declaration of Helsinki, and the complete study protocol was approved by the Max Stern Yezreel Valley College Ethics Review Board. Written informed consent was obtained from each participant and every participant was given $125 compensation for taking part in the study.

#### Sample size considerations

As our model included two groups and three observed variables, a sample size of 30 (15 per group) was necessary, assuming a medium effect size (0.06) at 95% power, *p* < 0.05, and r = 0.8 correlation between measurements.

### Measures

*The ADHD assessment questionnaire*, based on that developed by DuPaul and colleagues^29^, consists of 18 items based on the DSM–IV ADHD diagnosis symptoms, with nine items assessing hyperactivity and impulsivity, and nine items assessing attentiveness. In the version used in the current study, participants were asked to choose whether each described situation was correct or incorrect with respect to themselves. Scores ranged from 0 to 9 on each of the two scales, with one point for each ADHD symptom. This questionnaire has been used to screen for ADHD in many studies (e.g.^17,33^). The internal consistency (Cronbach’s α) of the attentiveness section and of the hyperactivity-impulsivity section of the scale in the current study were 0.92 and 0.83, respectively. The DSM–V suggests that in individuals aged 17 and over the appearance of at least five symptoms (i.e. scoring 5 on this questionnaire) is sufficient for an ADHD diagnosis. However, in order to guarantee that the ADHD group in this study included only participants with this condition, we limited participation to individuals with six or more symptoms.

*The Symptom Checklist-90 *(*SCL-90*^34^) was used to assess distress symptom occurrence rate with the purpose of excluding participants with psychiatric disturbances other than ADHD from the study. The SCL-90, completed during the screening phase of the experiment, is a 90-item, 5-point Likert scale questionnaire that measures the nine primary symptom dimensions of somatization, obsessive–compulsive, interpersonal sensitivity, depression, anxiety, hostility, phobic anxiety, paranoid ideation, and psychoticism. Patients are asked to rate the degree to which they have experienced symptoms within the last seven days. The retest reliability (correlation coefficients) of the symptom dimensions scales is r = 0.7–0.9. The SCL-90 has been translated into many languages and serves as an international standard.

*The structured clinical interview*, using a modified version of the DISC-IV^30^ ADHD module, yields clinician-assessed symptom counts for hyperactive-impulsive and inattentive ADHD symptoms. Interviews were conducted by educational psychology graduate students extensively trained on DISC-IV administration and scoring, with scoring supervised by a senior clinical psychologist. The DISC-IV scoring algorithm determines the presence or absence of ADHD based on DSM-IV symptom count, age of onset, impairment, and other parameters (see Bart et al.^35^ for more information).

*Actigraph sleep recordings* were performed over five days using an actigraph (Mini Motionlogger, Ambulatory Monitoring Inc., New York), a wrist-worn ambulatory device that uses a piezoelectric element to measure wrist movements. The actigraph samples wrist activity levels at 10-s intervals, sums those across 1-min intervals, and translates the result into 1-min long epochs of sleep and wake. Additionally, participants were instructed to press a button on the actigraph when they began trying to fall asleep (determining bedtime), and when they woke in the morning (determining wake time), and to also keep a sleep diary documenting their bedtimes and morning wake-up times. Raw data was processed using the Actigraphic Scoring Analysis program (W2 scoring algorithm) provided by the manufacturer to yield four sleep measures: total sleep time, sleep onset latency, wake time after sleep onset, and sleep efficiency (percentage of total sleep time between sleep onset and final awakening). Daily actigraphy data were averaged to yield aggregated measures for each participant.

*The Pittsburgh Sleep Quality Index (PSQI)* is an established tool for assessing subjective sleep quality^36^. The global PSQI score is determined by summing 18 items evaluating different aspects of sleep quality. A global PSQI score > 5 has been demonstrated to yield 89.6% diagnostic sensitivity and 86.5% specificity in distinguishing good and poor sleepers^36^, and thus can be considered a cut-off for clinically relevant sleep difficulties. The internal consistency (Cronbach’s α) of the PSQI was 0.66 in the current study.

*The Visual Oddball Task* The stimuli were three geometric shapes (triangle, square, circle) and photographs of three different male individuals’ faces. The set of photographs, taken from the standard NimStim face stimulus set^37^ of pictures of facial affect, showed each individual with an angry (target) and neutral (non-target) expression. Geometric shapes either had a black cross in the middle of the shape (target), or were “empty” (non-target). We used a four-stimulus visual oddball paradigm in which participants were shown regularly repeated standard stimuli (neutral faces and empty shapes) with 0.75 probability, and two “target” deviant stimuli (angry faces and shapes with a cross) with 0.25 probability. Each target stimulus was shown 10 times, and each non-target stimulus 30 times, resulting in a total of 240 stimuli (30 angry faces, 30 shapes with a cross, 90 neutral faces, 90 empty shapes). Stimulus presentation order was randomized between trials. In each trial, stimuli were displayed for 500 ms, followed by a 1000 ms inter-trial interval in which a blank screen was shown. The experiment, began with a short 32-trial practice block which contained stimuli corresponding to those presented in the experimental block. Similar oddball tasks have demonstrated high test–retest reliability^35–38^, and performing an oddball task several times in the same night was not shown to yield practice effects^21^.

### Procedure

As we have previously described^22,24,39^, for the five days prior to the experimental trial, participants were instructed to go to bed every night between 23:00 and 24:00, wake up between 7:00 and 9:00, sleep at least seven hours per night, refrain from napping, and avoid drinking more than three caffeinated beverages per day. In order to monitor their sleep during this period and ensure that they went to bed as instructed, the participants wore an actigraph on each night. The experimental session began at 8 am of Day 6 and lasted until ~ 10:30 am on Day 7, during which time the participants (four to eight participants in each session) were kept continuously awake (> 25 h).

At the beginning of the session the participants completed the PSQI, performed the visual oddball task (9:00–10:30 AM), and were then kept awake in the laboratory until they performed the visual oddball task again at 9:00 AM the next day. As previously described^17,40,41^, during every visual oddball task session, each participant sat 80 cm from a 19-inch computer screen. The participant was instructed to focus his gaze on the stimuli to be presented at the center of the screen and, as quickly as possible without compromising accuracy, to indicate the occurrence of a “target” (deviant) stimulus by pressing the computer keyboard’s spacebar with his right index finger, while withholding response to the “non-target” (standard) stimuli. Reaction time and error rate were recorded. There were two categories of error: omission (i.e., did not press the spacebar when a deviant stimulus appeared) and commission (i.e., pressed the spacebar when a standard stimulus appeared).

Responses could be made during stimulus presentation as well as during inter-trial intervals.

As we have previously described^22,24,29^, participants wore actigraphs on their wrist throughout the entire experimental session to verify that they remained awake and that they stayed under the laboratory’s controlled conditions (25 °C, ~ 500 lx lights on continually, sunlight undetectable). Participants did not smoke or consume food or beverages containing caffeine or cocoa, and were served organized meals at scheduled times under supervision. The participants were not permitted to perform any strenuous activity throughout the session, so when not being tested, they engaged in activities such as socializing and board games; participants of both study groups were involved equally in each of these activities. Experimental personnel present throughout the entire session ensured that no participant fell asleep. As this experiment was part of a larger study, participants’ emotional state and sleepiness were also tested throughout the experimental session.

### Data analysis

Before conducting any parametric tests, the normality assumption was examined (skewness and kurtosis between − 2 and + 2). Differences between the groups in the demographic variables and the pre-experimental sleep measures were examined using two-tailed independent samples t-tests or chi-squared tests. As multiple t-tests comparisons were performed, significance (*p* < 0.05) was determined following Bonferroni correction. These analyses were conducted using SPSS 25 software (SPSS Inc.).

In order to examine the effects of sleep deprivation on the responses of participants with and without ADHD to target faces versus target shapes (Hypothesis 1), we sought to perform a 2 × 2 × 2 mixed analyses of variance (ANOVA) on each of the four outcome variables (omission errors, commission errors, reaction time [RT], and RT standard deviation, [RTSD]). As these outcome variables of the oddball tests did not meet the normality assumptions, the aligned rank transform (ART) for nonparametric factorial ANOVAs^42^ was conducted. The ART relies on a preprocessing step that "aligns" data before applying averaged ranks, after which point ANOVA procedures can be used.

In each analysis the between-subject variables were time (baseline [time 0]/25 h into the experimental session [0 + 25]) target faces/shapes (for analysis of omission errors rates, RT, and RTSD) or non-target faces/shapes (for analysis of commission error rates) and group (ADHD/control), and the participant added as within-subject variable. These analyses were conducted using version 2.1.0 of ARTool in R environment (R version 4.0.5). No three-way interaction was detected (see below). Significant time × group interactions were further analyzed by conducting Wilcoxon signed-rank test in each of the study groups (ADHD and control).

In order to examine the interaction between target type (faces/shapes) and group (ADHD/control) at baseline (Hypothesis 2), mixed-model ANOVAs were conducted using the ART for nonparametric factorial ANOVAs. The between-subject variables were target faces/shapes (for analysis of omission errors rates, RT, and RTSD) or non-target faces/shapes (for analysis of commission error rates) and group (ADHD/control), and the participant added as within-subject variable. The significance threshold for all tests was set at *p* < 0.05.

## Results

Demographic comparisons between young adults with and without ADHD are presented in Table 1. The groups did not differ in age, caffeine and alcohol consumption, or proportion of cigarette smokers nor in their objectively measured sleep patterns. Notably, the average global PSQI score (a subjective measure of sleep quality) of the ADHD group was above 5, which is the cut-off for clinically relevant sleep difficulties. However, the groups did not differ significantly in their PSQI scores or in the percentage of participants receiving a global PSQI score above 5.

**Table 1:** Characteristics of the study samples.

	ADHD (N = 19)		Control (N = 16)		t	p
	Mean (SD)	Range	Mean (SD)	Range		
Age	24.7 (5.3)	18–36	26.3 (3.0)	19–30	1.13	0.27
ADHD-RS	14.5 (6.8)	6–27	0.8 (1.5)	0–5	7.**67**	< 0.001*
Alcohol	0.4 (0.6)	0–2	0.5 (0.8)	0–3	0.45	0.65
Caffeine	2.3 (0.9)	0–3	2.2 (0.8)	1–3	0.26	0.80
Smokers (%)	40.0		18.8		χ^2^ = 1.70	0.25
Total sleep time (min.)	423.1 (47.7)	332.2–501.2	426.0 (49.9)	335.6–519.8	0.16	0.87
Sleep onset latency (min.)	27.8 (33.7)	2.0–100.0	19.3 (12.8)	4.1–41.0	0.96	0.35
Sleep efficiency (%)	91.5 (8.2)	66.4–99.7	96.2 (4.4)	82.7–99.7	1.95	0.06
WASO (min.)	25.8 (19.4)	1.4–62.9	15.0 (17.4)	1.5–68.0	1.61	0.12
Global PSQI score	6.0 (3.0)	1–11	4.4 (2.9)	0–11	1.52	0.14
PSQI > 5 (%)	52.9		31.3		χ^2^ = 1.59	0.30

Demographic characteristics and subjective measures of the participants’ sleep prior to the experimental session. *SD* Standard deviation, *ADHD-RS* ADHD Rating Scale, *Alcohol* number of alcoholic beverages per week, *Caffeine* number of caffeinated beverages per day, *WASO* wake after sleep onset, *PSQI* Pittsburgh Sleep Quality Index. Differences between the groups in the demographic variables and the pre-experimental sleep measures were examined using two-tailed independent-samples t-tests or chi-squared tests. As multiple t-test comparisons were performed, significance (*p < 0.05) was determined following the Bonferroni correction.

Descriptive statistics of the omission errors, commission errors, RTs, and RTSDs in response to face and shape targets among the ADHD and control groups before and after sleep deprivation are presented in Table 2.

**Table 2:** Measures of the oddball task: descriptive statistics.

	ADHD (N = 19)				Control (N = 16)			
	Mean (SD)	Range	Mean (SD)	Range	Mean (SD)	Range	Mean (SD)	Range
**Facial stimuli**								
Omission errors	0.4 (0.8)	0–3	3.4 (4.8)	0.0–17.0	0.4 (0.7)	0–2	0.4 (0.5)	0–1
Commission errors	1.4 (1.2)	0–5	3.4 (3.9)	0.0–13.0	1.6 (1.7)	0–6	1.7 (2.4)	0–8
RT (ms)	487.8 (48.2)	426.0–607.4	498.6 (107.2)	343.5–804.7	475.2 (49.7)	401.8–560.7	513.6 (43.3)	419.9–563.8
RTSD	105.9 (48.9)	45.4–208.8	214.4 (114.3)	45.0–428.5	97.4 (37.3)	51.0–165.2	119.4 (51.4)	54.4–217.4
**Non-facial stimuli**								
Omission errors	0.0 (0.0)	0–0	2.7 (4.2)	0.0–12.0	0.0 (0.0)	0 -0	0.9 (2.1)	0–8
Commission errors	0.4 (0.7)	0–2	2.7 (3.2)	0.0–10.0	0.2 (0.4)	0–1	0.5 (0.9)	0–0
RT (ms)	423.6 (45.8)	373.6–526.9	422.0 (81.6)	302.5–560.3	426.5 (35.4)	375.5–492.1	450.0 (65.7)	396.5–663.0
RTSD	76.5 (32.4)	38.3–142.4	152.2 (85.9)	43.3–298.6	80.3 (43.0)	37.0–176.9	78.9 (44.1)	0.0–155.8

Performance of the ADHD and control group participants in response to target faces/shapes (omission errors, reaction time [RT], and reaction time variability [RT standard deviation, RTSD]) and non-target faces/shapes (commission errors) before (time 0, baseline) and after (0 + 25) sleep deprivation.

### Effects of sleep deprivation

#### Omission errors (Fig. 1)

**Figure 1:**
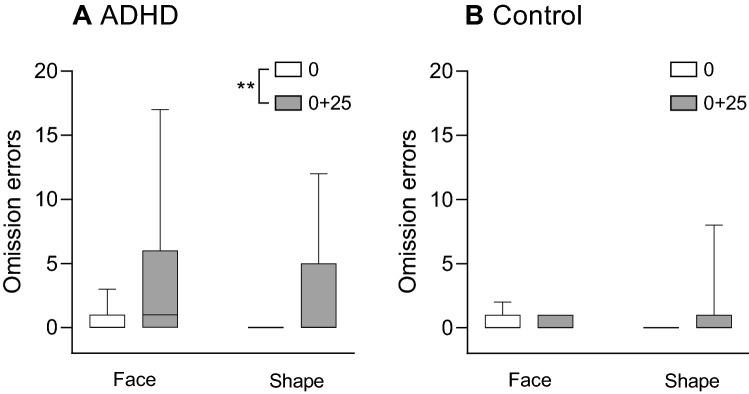
Boxplot (median, minimum–maximum) of the rates of omission errors on trials with target (angry) faces or target shapes for the ADHD group (A) and the control group (B). Differences between the rates of omission errors before (time 0) and after sleep deprivation (time 0 + 25) were examined across target type for each group using the Wilcoxon signed-rank test. **p < 0.01.

A 2 × 2 × 2 mixed nonparametric ANOVA was conducted, with the between-subject variables being time (baseline/time 0 + 25), target type (face/shape) and group (ADHD/control), and the participants being the within-subject variable. The analysis revealed no significant three-way interaction (F_(1,99)_ = 1.33, *p* = 0.251). However, there was a main effect for time (F_(1,99)_ = 38. 63, *p* < 0.001), indicating that there were more errors following sleep deprivation than at baseline. There was, in addition, a significant time × group interaction (F_(1,99)_ = 19.03, *p* < 0.001). To analyze this interaction, a Wilcoxon signed-rank test was conducted on each of the study groups, revealing that the increase in omission errors following sleep deprivation was significant in the ADHD group (Z = − 2.80, *p* = 0.005) but not in the control group (Z = − 1.69, *p* = 0.0.90).

There was also a main effect for group (*F*_(1,33)_ = 3.92, *p* = 0.012), indicating that there were more omission errors in the ADHD group. Finally, there was no main effect for target type (*F*_(1,99)_ = 0.29, *p* = 0.589), no time × target type interaction (*F*_(1,99)_ = 2.46, *p* = 0.12), and no group × target type interaction (*F*_(1,99)_ = 1.05, *p* = 0.307).

#### Reaction time (RT; Fig. 2)

**Figure 2:**
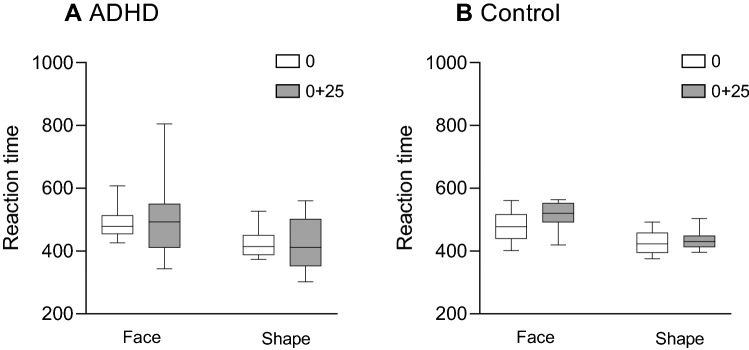
Boxplot (median, minimum–maximum) of the reaction times (RT) in response to target (angry) faces or target shapes for the ADHD group (A) and the control group (B).

Analysis of the reaction time in response to target faces and target shapes via a 2 × 2 × 2 mixed nonparametric ANOVA revealed no significant three-way interaction (*F*_(1,93)_ = 0.38, *p* = 0.563). There was a main effect for target type (*F*_(1,93)_ = 82.26, *p* < 0.001), indicating that reaction time was longer in response to non-target faces than in response to non-target shapes. However, there was no significant main effect for time (*F*_(1,93)_ = 3.59, *p* = 0.061) or for group (*F*_(1,31)_ = 0.35, *p* = 0.560). Similarly, there was no significant time × group interaction (*F*_(1,93)_ = 1.33, *p* = 0.252), group × target type interaction (*F*_(1,93)_ = 0.13, *p* = 0.721), or time × target type interaction (*F*_(1,93)_ = 1.50, *p* = 0.224).

#### Reaction time variability (RTSD; Fig. 3)

**Figure 3:**
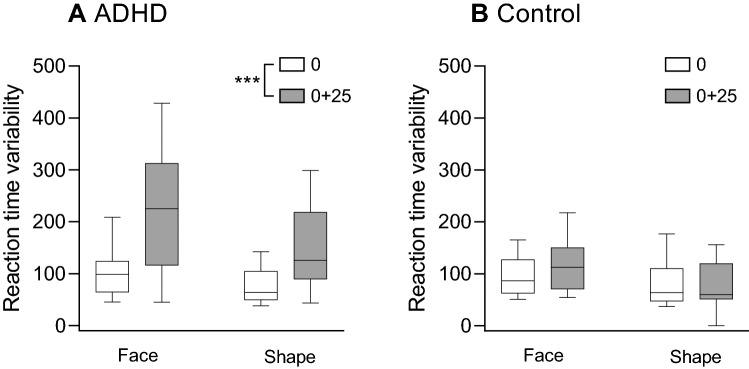
Boxplot (median, minimum–maximum) of the reaction time variability (RTSD) on trials with target faces or target shapes for the ADHD group (A) and the control group (B). Differences between the rates of omission errors before (time 0) and after sleep deprivation (time 0 + 25) were examined across target type for each group using the Wilcoxon signed-rank test. ***p < .001.

Analysis of the reaction time variability in response to target faces and target shapes via a 2 × 2 × 2 mixed nonparametric ANOVA revealed no significant three-way interaction (*F*_(1,93)_ = 0.87, *p* = 0.354). However, there was a main effect for time (*F*_(1,93)_ = 43.06, *p* < 0.001), indicating that RTSD increased following sleep deprivation. In addition, there was a significant time × group interaction (*F*_(1,93)_ = 25.11, *p* < 0.001). To analyze this interaction, a Wilcoxon signed-rank test was conducted on each of the study groups, revealing that the increase in RTSD following sleep deprivation was significant in the ADHD group (Z = − 3.38, p < 0.001) but not in the control group (Z = − 0.73, p = 0.460).There was also a main effect for group (*F*_(1,31)_ = 9.84, *p* = 0.004), indicating that RTSD in response to targets was higher in the ADHD group. There was also a main effect for target type (*F*_(1,93)_ = 29.60, *p* > 0.001), indicating that RTSD was higher in response to target faces rather than to target shapes. Finally, there was no group × target type interaction (*F*_(1,93)_ = 1.12, *p* = 0.293).

#### Commission errors (Fig. 4)

**Figure 4:**
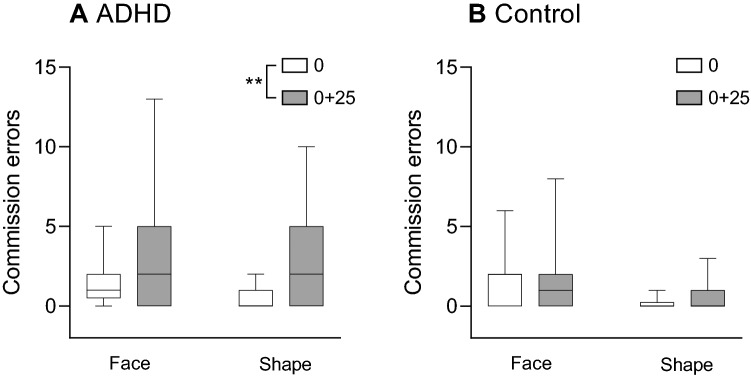
Rates of commission errors on trials with non-target faces or non-target shapes for the ADHD group (A) and the control group (B). Differences between the rates of omission errors before (time 0) and after sleep deprivation (time 0 + 25) were examined across target type for each group using the Wilcoxon signed-rank test. **p < 0.01.

Analysis of the commission errors in response to non-target faces via a 2 × 2 × 2 mixed nonparametric ANOVA revealed no significant three-way interaction (*F*_(1,99)_ = 0.04, *p* = 0.841). However, there was a main effect for time (*F*_(1,99)_ = 21.17, *p* < 0.001(, indicating that there were more errors following sleep deprivation than at baseline. In addition, there was a significant time × group interaction (*F*_(1,99)_ = 12.76, *p* < 0.001). To analyze this interaction, a Wilcoxon signed-rank test was conducted on each of the study groups, revealing that the increase in commission errors following sleep deprivation was significant in the ADHD group (Z = − 3.01, *p* = 0.003) but not in the control group (Z = 0.0, *p* > 0.999).

There was also a main effect for target type (*F*_(1,99)_ = 13.98, *p* < 0.001), indicating that there were more commission errors in response to non-target faces rather than non-target shapes. There was also a main effect for group (*F*_(1,33)_ = 6.31, *p* = 0.017). Finally, there was no group × target type interaction (*F*_(1, 99)_ = 0.01, *p* = 0.918), or time × target type interaction (*F*_(1,99)_ = 0.36, *p* = 0.552).

### Oddball results at baseline (the morning beginning the session; time 0)

Mixed nonparametric ANOVAs on omission and commission errors at baseline revealed a main effect for target type (faces/shapes) (omission: *F*_(1,33)_ = 6.54, *p* = 0.015; commission: F_(1,33)_ = 28.34, *p* < 0.001). The rates of omission errors in response to target faces was higher than in response to target shapes, and the rates of commission errors in response to the non-target faces higher than in response to the non-target shapes. There was no main effect for group (omission: *F*_(1,33)_ = 0.99, *p* = 0.330; commission: *F*_(1,33)_ = 0.42, *p* = 0.522) or target type × group interaction (omission: *F*_(1,33)_ = 0.94, *p* = 0.340; commission: *F*_(1,33)_ = 0.09, *p* = 0.767).

Similarly, mixed nonparametric ANOVAs on the RT and RTSD at baseline revealed a main effect for target type (faces/shapes), with longer mean RT (*F*_(1,32)_ = 100.68, *p* < 0.001) and higher mean RTSD (*F*_(1,32)_ = 14.11, *p* < 0.001) in response to target faces than in response to target shapes. There was no main effect for group (RT: *F*_(1,32)_ = 0.01, *p* = 0.920; RTSD: *F*_(1,32)_ = 0.23, *p* = 0.633) or target type × group interaction (RT: *F*_(1,32)_ = 2.39, *p* = 0.132; RTSD: *F*_(1,32)_ = 0.038, *p* = 0.848).

## Discussion

To the best of our knowledge, this study is the first study to compare young adults with and without ADHD in terms of the effects among young adults with and without ADHD of prolonged acute sleep deprivation (25 h of sustained wakefulness) on the processing of emotional facial expressions (angry faces) and non-facial stimuli (geometric shapes) as reflected in the visual oddball paradigm. Specifically, the visual oddball paradigm examines the process that allows for the maintenance of response persistence over time (i.e., sustained attention) to visual stimuli, with decreased sustained attention reflected by lower reaction time to the stimuli (RT), instability in the reaction time to the stimuli (RTSD), and difficulties in detecting the target stimuli and in response inhibition to the non-target stimuli.

Our first hypothesis was that following sleep deprivation, performance on the oddball task would deteriorate, as reflected by lower sustained attention (higher rates of omission errors, longer RTs, and higher RTSD) and decreased response inhibition (higher rates of commission errors). We expected the level of deterioration to be higher in the ADHD group than the control group and in response to facial rather than non-facial stimuli. This hypothesis was partly confirmed. As hypothesized, sleep deprivation had a greater effect on the performance of the ADHD group than on the control group. Specifically, as reflected by significant time × group interactions, sleep deprivation significantly increased the rates of omission errors and commission errors, and the levels of RTSD, in the ADHD group but not in the control group. Sleep deprivation, however, did not significantly affect RT to the target stimuli, which may imply that sleep deprivation increases instability in the reaction time to visual stimuli without causing an overall increase in the reaction times. In this regard, it is noteworthy that the lack of difference between the RTs of the ADHD group and the RTs of the control group is in line with several meta-analyses reporting that the RTs of individuals with ADHD are not necessarily slower than those of individuals without ADHD but tend to be far less stable^43^.

The finding that sleep deprivation has deleterious effects on sustained attention and response inhibition is consistent with previous controlled studies conducted among young adults^22^ and children^20^ and with reports stating that sleep difficulties aggravate impulsivity and inattentiveness in children and adults with ADHD^26,27^. However, the finding that ADHD is associated with greater sensitivity to the negative effects of sleep deprivation on sustained attention and response inhibition is novel. In previous studies, which used continuous performance tests (CPT), sleep deprivation was found to increase omission errors, commission errors, and RTSD among participants with and without ADHD, with no significant differences between the groups in terms of the magnitude of this increase^22^. The reason for the disparity between the results of these studies and the current study is likely due to the differences between the CPT used in the former and the oddball task used here. Most significantly, the CPT task comprised of one circle and one triangle, whereas the oddball task included a more complex set of stimuli: two facial expressions and different geometric shapes. It is possible that following sleep deprivation, individuals without ADHD fail to sustain attention when engaged in a simple and monotonous task but a more challenging and demanding task prompts them to employ their attentional resources. Individuals with ADHD, on the other hand, fail to sustain attention following sleep deprivation regardless of such characteristics of the task.

The reasons for the heightened vulnerability of individuals with ADHD to the effects of sleep deprivation on sustained attention are unclear but possibly involve deficits in brain systems that are involved in attention and response inhibition and are sensitive to the effects of sleep deprivation. Specifically, sleep deprivation attenuates activation of various parietal and frontal cortical regions, including the prefrontal cortex^44,45^, which plays a central role in sustained attention. Notably, ADHD is associated with lower activation in the prefrontal cortex during tasks that involve attention^46^ and inhibitory control tasks^47^. Thus, sleep deprivation may aggravate the deficits in the functioning of the prefrontal cortex and related regions, leading to a more severe disruption in the performance of tasks that require sustained attention.

As expected, participants with and without ADHD demonstrated slower RTs, and greater RTSD (but not a significantly higher rate of omission errors) in response to facial targets than in response to non-facial targets and more commission errors in response to facial non-targets than in response to non-facial targets on baseline as well as following sleep deprivation. The findings regarding RTs, RTSD, and commission errors are consistent with previous studies^17^ and may be attributed to the greater complexity and/or added emotional content of facial stimuli.

However, in contrast to our hypothesis, the lack of time × group × target type interactions and time × target type interactions indicates that sleep deprivation had a similar effect on participants’ sustained attention capabilities and response inhibition regardless of whether facial or non-facial stimuli served as targets and non-targets. It can thus be concluded that for individuals with ADHD sleep deprivation affects the sustained attention to visual stimuli in general and not to processes that are specific to facial features or the emotional content of facial expressions.

Our second hypothesis was that prior to sleep deprivation (i.e., baseline) participants with ADHD would perform worse than controls in the measures of the oddball task. There were, however, no differences between the groups in any of these measures. This result contrasts with previous studies which demonstrated that adults with ADHD perform more poorly than adults without ADHD in similar tasks that require sustained attention and response inhibition^17,21,38^. In particular, Raz and Dan^17^ used the same protocol as the current study (but without sleep deprivation) and showed that participants with ADHD had higher rates of omissions, slower RTs, and higher RTSDs in response to facial and non-facial targets than participants without ADHD. The main difference between the current study and similar previous studies was that the current study attempted to control for the participants’ sleep quality and quantity at baseline. This was done because individuals with ADHD tend to suffer more than individuals without ADHD from poor sleep and excessive daytime sleepiness^19,48,49^, with the most common sleep difficulty associated with ADHD being a delayed sleep/wake cycle^50–52^, resulting in shorter sleep duration and accumulated sleep propensity^48^. We therefore limited participation in the current study to people without known sleep disorders, and the participants were instructed to go to bed every night between 23:00 and 24:00, wake up between 7:00 and 9:00, and sleep at least seven hours per night for the five days prior to the experimental trial. Adherence to these instructions were verified using actigraphy. It is therefore possible that deficits in sustained attention and response inhibition among individuals with ADHD are due, at least partially, to sleep difficulties and conditions of insufficient sleep. This possibility is supported by studies demonstrating that individuals with ADHD not only tend to suffer more than individuals without ADHD from poor sleep and excessive daytime sleepiness^19,49^ but are also more sensitive to the effects of sleep deprivation on sleepiness^39^. Sleep deprivation and/or increased sleepiness have been shown to be associated with impaired cognitive functioning^53^, reduced ability to accurately identify emotional expressions^23,54^, and functional decline in brain areas involved in conscious visual cognition of facial expressions^55^.Taken together, the findings of the current study suggest that people with ADHD are more sensitive to the effects of sleep deprivation on sustained attention and cognitive functions that rely on attention, including the accurate identification of emotional facial expressions. Given that ADHD is associated with poor sleep and excessive daytime sleepiness^19,48^, it is feasible that poor sleep quality and quantity play an important role in the difficulties in sustained attention and, consequently, in the ability to accurately attend to emotional facial expressions that characterize many individuals with ADHD.

The finding that sleep deprivation hinders the ability of individuals with ADHD to sustain attention to visual stimuli, including emotional facial expressions, has important clinical implications. First, it implies that insufficient sleep, whether due to sleep difficulties or an irregular sleep schedule, may impair the cognitive functioning of individuals with ADHD to a greater degree than individuals without ADHD. This has obvious negative consequences on academic and professional performance and an increased risk of accidents. Second, emotional facial expressions are a critical cue for accurately perceiving others’ emotions, which is essential for adequate social interactions^8,53^. Increased difficulties in the interpretation of emotional facial expression due to deficits in sustained attention resulting from insufficient sleep may contribute to the social, interpersonal, and emotional difficulties that are associated with ADHD in adulthood^54,55^. Hence, ensuring sufficient sleep and avoiding conditions of sleep deprivation may be critical elements in ADHD treatment. Supporting this assertion, Lopez et al.^56^ reviewed 14 randomized controlled trials evaluating the effects of cognitive-behavioral therapies for insomnia (CBT-I) on adults with ADHD, demonstrating that these interventions consistently reduced the severity of core ADHD symptoms.

The current study has several limitations. First, only one emotional facial expression (anger) was used. Previous studies have shown that the existence or the gravity of deficits in emotional facial expression processing (associated with ADHD or sleep deprivation) may depend on the type of emotion expressed^57,58^. Thus, more research is needed to determine the effects of sleep deprivation on the processing of facial expressions other than anger in individuals with ADHD. Second, only young men were included in the study in order to lower variability and assure adequate statistical power for examining the study’s main hypotheses. Thus, generalization of the findings to women and other age groups should be made with caution. Third, 25 h of sustained wakefulness is an extreme condition of sleep deprivation which is not commonly experienced by young adults. A much more common condition among this population is chronic insufficient sleep^59^. Thus, additional examination is required to elucidate whether such limited sleep deprivation produces the effects observed in the current study. Fourth, our exclusion of participants with periodic limb movements in sleep, obstructive sleep apnea, or restless legs syndrome was based on subjective self-reports. A more definite elimination of such disorders requires objective tests, such as polysomnography, which were not conducted in the current study. Fifth, it could be argued that it was the repetition of the oddball task rather than the lack of sleep that affected the performance of the participants. However, this is unlikely as similar oddball tasks have demonstrated high test–retest reliability with no indication of a practice effect among participants with and without ADHD^35,38^. In particular, in Sedah et al.’s study^21^, participants performed a similar oddball task once in the morning and once in the evening with the order being counter balanced. The results indicated a good test–retest reliability and no notable differences between the two trials. Finally, during the 24 h preceding the experimental session, the participants of the ADHD group did not take ADHD stimulant medications (a washout period was used for such medications in previous studies^32,60^). Thus, rebound effects or withdrawal symptoms might have affected the performance of some participants. Nevertheless, only limited, if any, rebound or withdrawal effects among adult ADHD patients were reported in studies examining the effects of discontinuation of treatment with such medications^61,62^.

In conclusion, the current study suggests that sleep deprivation has a detrimental effect on sustained attention to visual stimuli, including emotional facial expressions, of young male adults with ADHD. These results further support the proposition that sleep quantity and quality have a substantial effect on the severity of ADHD symptoms.

## Data Availability

The datasets generated and analyzed during the current study are available from the corresponding author on reasonable request.
